# Case report: A case of acute disseminated encephalomyelitis after SARS-CoV-2 infection in pediatric patients

**DOI:** 10.3389/fneur.2023.1099458

**Published:** 2023-02-22

**Authors:** Francesca Cautilli, Mariavittoria Feleppa, Massimiliano Valeriani, Laura Papetti, Gabriele Monte, Fabio Midulla, Alberto Spalice

**Affiliations:** ^1^Child Neurology Division, Department of Pediatrics, “Sapienza” University of Rome, Rome, Italy; ^2^Developmental Neurology Unit, Bambino Gesù Children's Hospital, IRCCS, Rome, Italy; ^3^Department of Pediatrics, “Sapienza” University of Rome, Rome, Italy

**Keywords:** COVID-19, complications, neurological complications, children, acute disseminated encephalopathy

## Abstract

**Introduction:**

Since the beginning, there has been enough evidence about the multi-systematic involvement of the coronavirus disease 2019 (COVID-19), which is caused by severe acute respiratory syndrome coronavirus 2 (SARS-CoV-2). Recent observations have revealed that, together with others, typical neurological manifestations are also associated with COVID-19 infection. In the first 2 years, children accounted for a few percent of cases, but with the emergence of the Omicron variant, the number of cases in the pediatric population has increased. It has been described that ~5% of the affected population suffered from severe neurological complications, such as seizure, coma, encephalitis, demyelinating disorders, and aseptic meningitis. Acute disseminated encephalomyelitis (ADEM) is an inflammatory demyelinating disease of the central nervous system. Typically, it presents in childhood and occurs 1 or 2 weeks after infection or vaccination.

**Case presentation:**

We present the case of a 12-year-old boy who developed ADEM, 10 days after an asymptomatic SARS-CoV-2 infection. Neurological symptoms began with headache, fever, irritability, paraplegia, and loss of sensitivity from the T1 level. The diagnosis of ADEM was confirmed by the typical signs found on brain MRI, whereas spinal cord MRI showed signs of transverse myelitis. The cerebrospinal fluid (CSF) testing excluded infections and did not reveal oligoclonal antibody bands (anti-MOG-negative and anti-AQP-negative). High-dose steroids (30 mg/kg/day) and IVIG (2 g/kg) were administered to the patient without any clinical improvement. The patient received a cycle of plasma exchange therapy, followed by rituximab infusion, with partial improvement. After 3 months, the magnetic resonance imaging (MRI) results demonstrated radiological improvement in accordance with the ADEM diagnosis.

**Conclusion:**

This clinical case confirms that SARS-CoV-2 infections are increasingly implicated in severe neurological consequences in both adult and pediatric patients. While the most frequent complications that were reported in children included headache, altered mental status, and encephalopathy, ~5% of the individuals suffered from severe neurological complications, leading to lifelong sequelae. All physicians must be aware of these data and detect neurological signs of severe (or not) complications that require a specific follow-up and treatment.

## Introduction

Severe acute respiratory coronavirus 2 (SARS-CoV-2), which caused the coronavirus disease (COVID-19) pandemic, continues to spread worldwide. As of 7 October 2022, more than 621 million confirmed cases have been reported in the general population. In the first 2 years of the pandemic, children accounted for a small percentage of cases (<5%) (1). However, with the diffusion of the Omicron variant, a higher percentage of cases have been reported in children, with more than 59 million confirmed cases in the age group 0–19 years (2). Although infection in the pediatric population is associated with asymptomatic cases or less severe manifestations (fever, dry cough, flu, and fatigue) than that in adults, it was reported that up to 6% of patients have been reported to have severe manifestations (3). Moreover, it has been shown that patients who were hospitalized with severe disease and/or multi-systemic inflammatory syndrome in children (MIS-C) develop distinct neurological symptoms (acute or not) in 22% of the cases (4). Among these, ~5%−12% of the individuals developed life-threatening neurological diseases such as severe encephalopathy, ischemic or hemorrhagic stroke, acute central nervous system infection, acute disseminated encephalomyelitis (ADEM), acute fulminant cerebral edema, and Guillain–Barré syndrome (GBS). Approximately one-fourth of these patients were at risk of death, and one-third displayed some disability at the time of discharge from the hospital (4, 5). ADEM, also known as post-infectious encephalomyelitis and immune-mediated encephalomyelitis, is a multifocal and monophasic inflammatory demyelinating disease of the central nervous system (CNS). This involves multiple areas of the white matter and rarely involves gray matter and the spinal cord. This pathology typically affects children under 10 years of age, mostly boys with a medical history of viral or bacterial infections. On rare occasions, such pathology was witnessed in individuals who developed symptoms within 2–40 days of vaccination (6), with some cases of ADEM also after COVID-19 vaccination (7). Interestingly, during the COVID-19 pandemic, several cases of ADEM related to SARS-CoV-2 infection have been reported in adults, with a higher incidence than usual (9). Here, we present a case of a 12-year-old boy who developed ADEM after an asymptomatic SARS-CoV-2 infection and was referred to Bambino Gesù Children's Hospital (Rome, Italy) for definitive diagnosis and treatment.

## Case description

### Patient information

Our case is a 12-year-old boy who was transferred to the Bambino Gesù Children's Hospital from another hospital in March 2022, for the management and treatment of severe neurological symptoms. The symptoms began 10 days after asymptomatic SARS-CoV-2 infection. SARS-CoV-2 infection was diagnosed by a nasopharyngeal swab polymerase chain reaction (PCR) test, which was performed after exposition to the virus. Notable neurological symptoms included irritability, headache, and difficulty in urinating. At 2 days after the onset of symptoms, the patient presented with fever, paraplegia, and loss of sensitivity from the T1 level.

His medical history included growth retardation, genetic disorder, community-acquired pneumonia, and angioedema. The individual had been administered all the mandatory vaccines in Italy. However, the SARS-CoV-2 vaccine was not administered.

### Clinical findings

Upon admission to the hospital, the child was alert and interactive. There was no cranial nerve dysfunction. Lower tendon reflexes and superficial abdominal reflexes were absent. Loss of sensitivity from the T1 level and paraplegia was observed. Dysmetria of the extremities was not present. The patient presented with bladder overdistention and fever.

The cardiorespiratory system was not involved, and O_2_ saturation was 100% in room air, with stable and autonomous hemodynamics.

### Timeline

#### Diagnostic assessment

**Figure 1:**
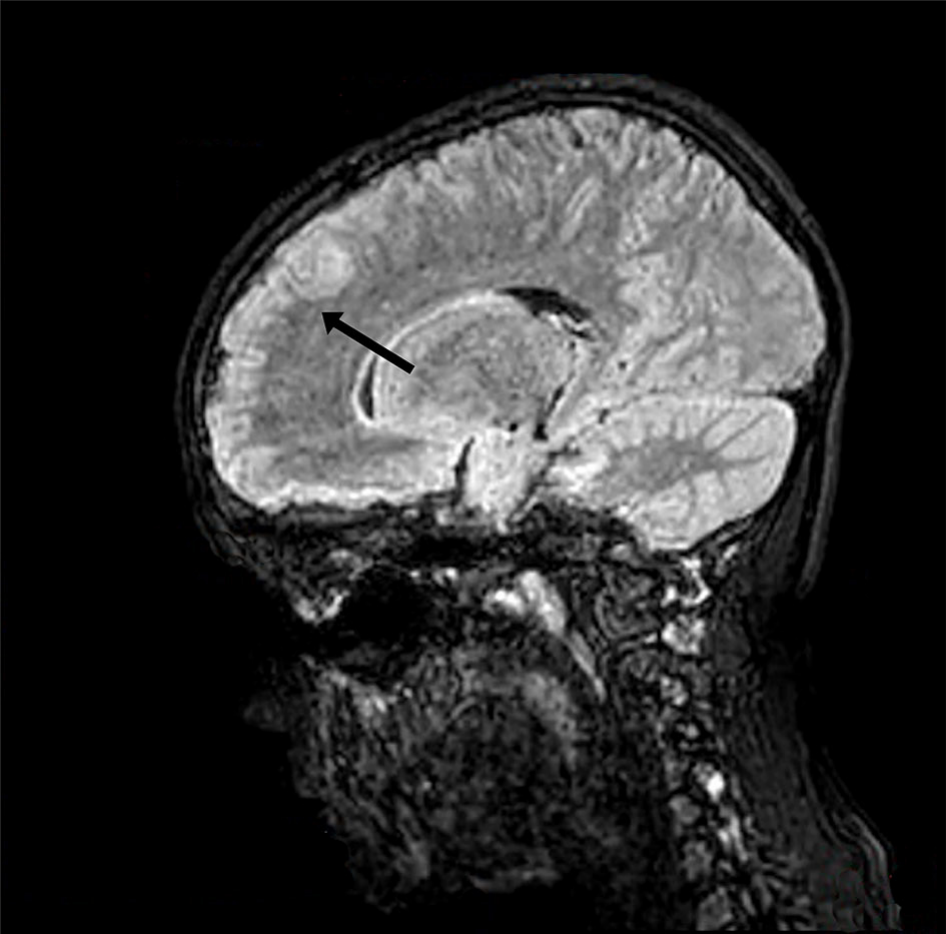
Three-dimensional (3D) brain MRI, brain sagittal image with signal alteration corresponding to the subcortical white substance.

**Figure 2:**
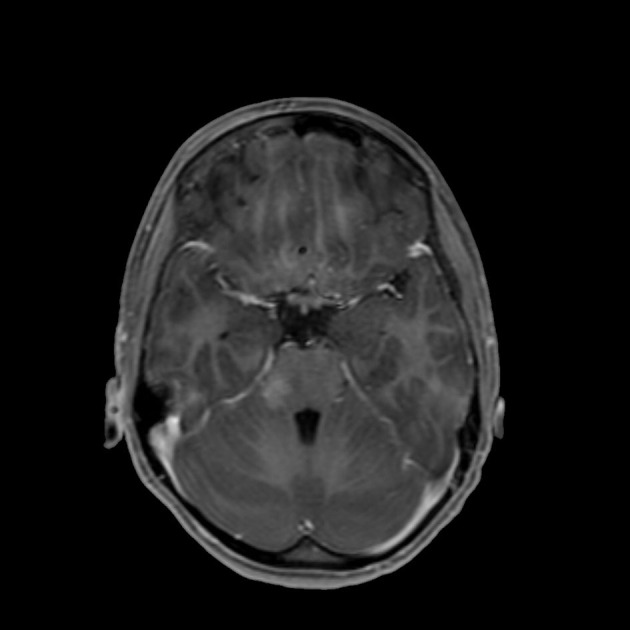
T1-weighted brain MRI, brain axial image with signal alteration in the subcortical white substance.

**Figure 3:**
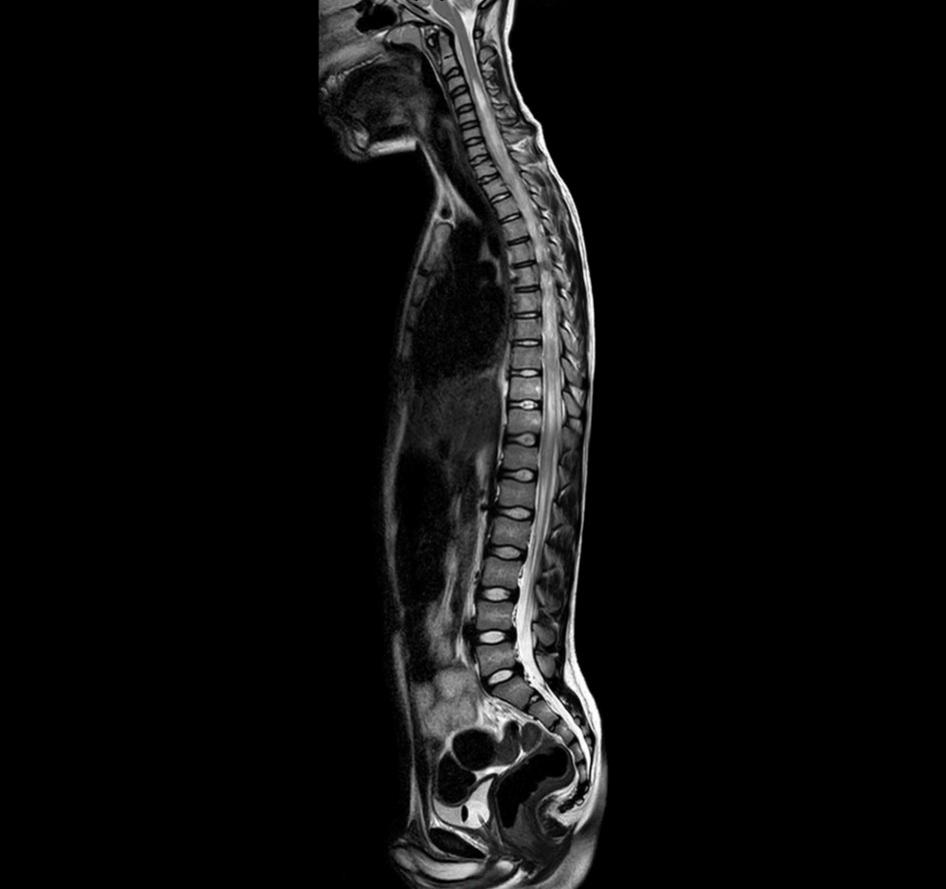
T2-weighted (T2W) turbo spin echo (TSE) magnetic resonance imaging (MRI), spinal cord sagittal image with signal alterations from C4 to the conus medullaris.

At the onset of symptoms, a computed tomography (CT) scan of the brain was performed to exclude neurosurgical emergencies. Brain and spinal cord MRI with contrast revealed “signal alterations corresponding to the pons, the right middle cerebellar peduncle, and the subcortical white substance of the frontal lobes; the alterations at the right middle cerebellar peduncle and pons enhanced after gadolinium” (Figures 1, 2); and “signal alterations from C4 to the conus medullaris, with contrast impregnation at D1 and conus medullaris level” (Figure 3). These images were compatible with that of ADEM with spinal cord involvement. The CSF testing excluded infections, and the result was compatible with an immune-mediated disease (51 mg/dl proteins, 59/mm^3^ white blood cells, and 6/mm^3^ red blood cells). CSF and blood cultures were negative.

Band autoantibodies were not detected in the CSF liquor and blood samples, which were anti-MOG-negative and anti-AQP4-negative (measured by enzyme-linked immunosorbent assay). Laboratory tests of blood samples excluded autoimmunity, infections, and immune deficiencies.

In particular, antinuclear antibodies (ANA), extractable nuclear antigens (ENA), anti-smooth muscle antibodies (ASMA), anti-parietal cell antibodies (APCA), endomysial antibodies (EMA IgA), anti-liver-kidney microsome antibodies (LKM), antibodies to double-stranded DNA (Anti Ds-DNA), anti-neutrophil cytoplasmic antibodies (c-ANCA and p-ANCA), anti-cardiolipin antibodies, anti-beta-2-glycoprotein antibodies, and anti-citrullinated protein antibodies (anti-CCP) were negative.

*Mycobacterium tuberculosis* DNA was not isolated; the nasopharyngeal swab was negative for respiratory viruses. Laboratory tests of blood samples were negative for other infections; in particular, cytomegalovirus (CMV), hepatitis B (antibodies positive for vaccination), hepatitis A (antibodies positive for vaccination), hepatitis C, *Treponema pallidum*, human immunodeficiency virus (HIV), and saccharomyces cerevisiae, were tested.

Lymphocyte subpopulations were normal.

Electroencephalography (EEG) was normal. The cardiological evaluation did not reveal any problems. Neurophysiological studies revealed normal visual evoked potentials (VEPs), whereas somatosensory evoked potentials (SSEPs) and motor evoked potentials (MEPs) were altered.

### Therapeutic intervention

High-dose steroids (30 mg/kg/day for 3 days) and intravenous immunoglobulin (IVIG; 2 g/kg/day) were administered to the patient without any clinical improvement. The patient received five cycles of plasma exchange therapy, after the parental declaration of informed consent, followed by off-label rituximab infusion (weekly infusion for 4 weeks) as reported in recent pediatric severe cases of ADEM (8). The patient showed partial clinical improvement along with important radiological improvement.

### Follow-up and outcomes

**Figure 4:**
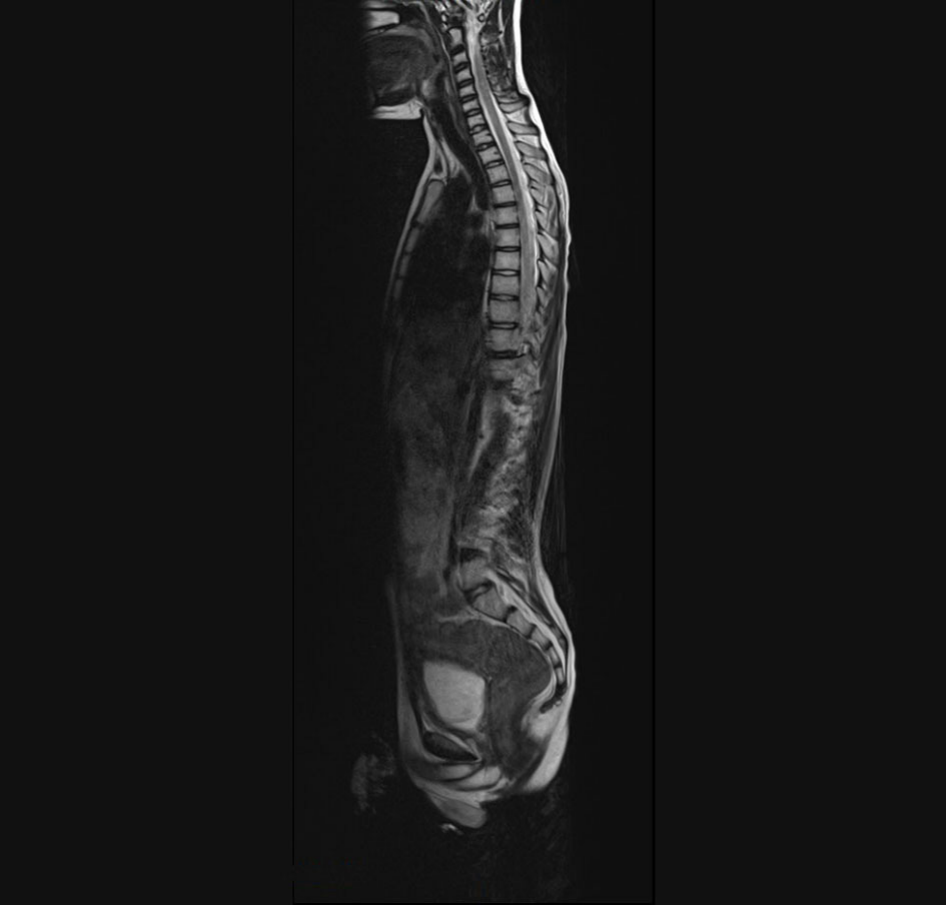
T2 MRI, spinal cord sagittal image without alterations.

**Figure 5:**
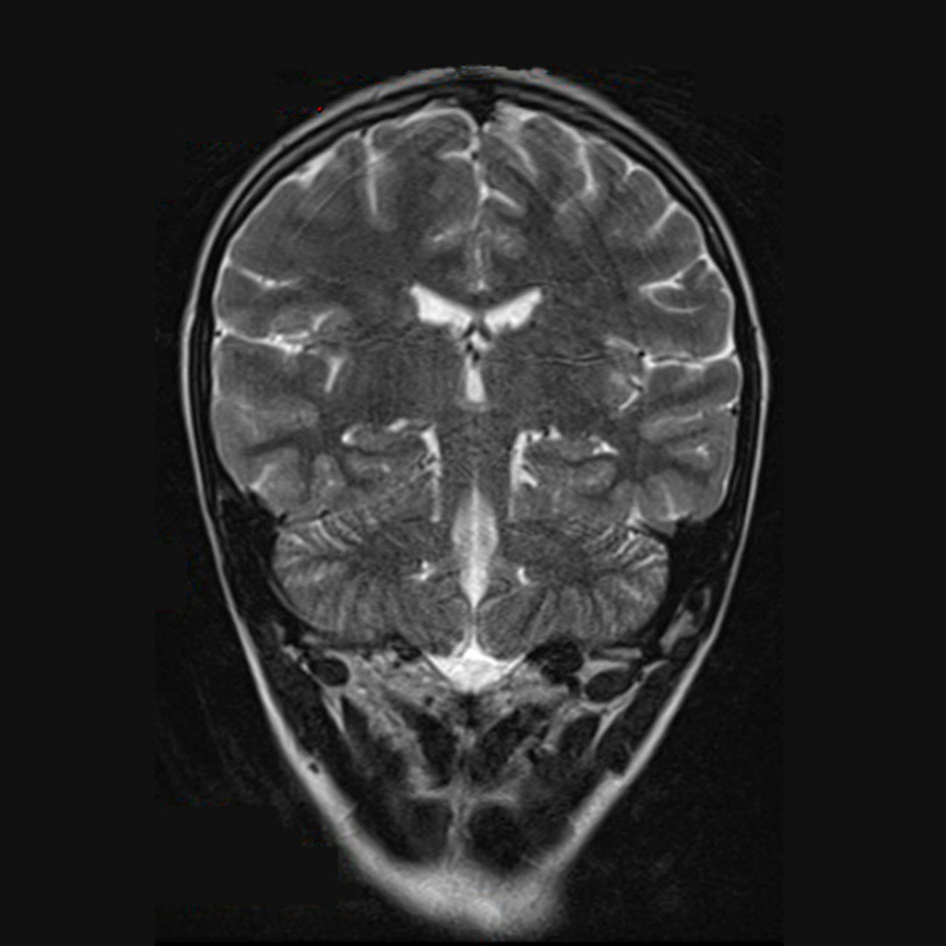
T2 MRI, brain coronal image without alterations.

**Figure 6:**
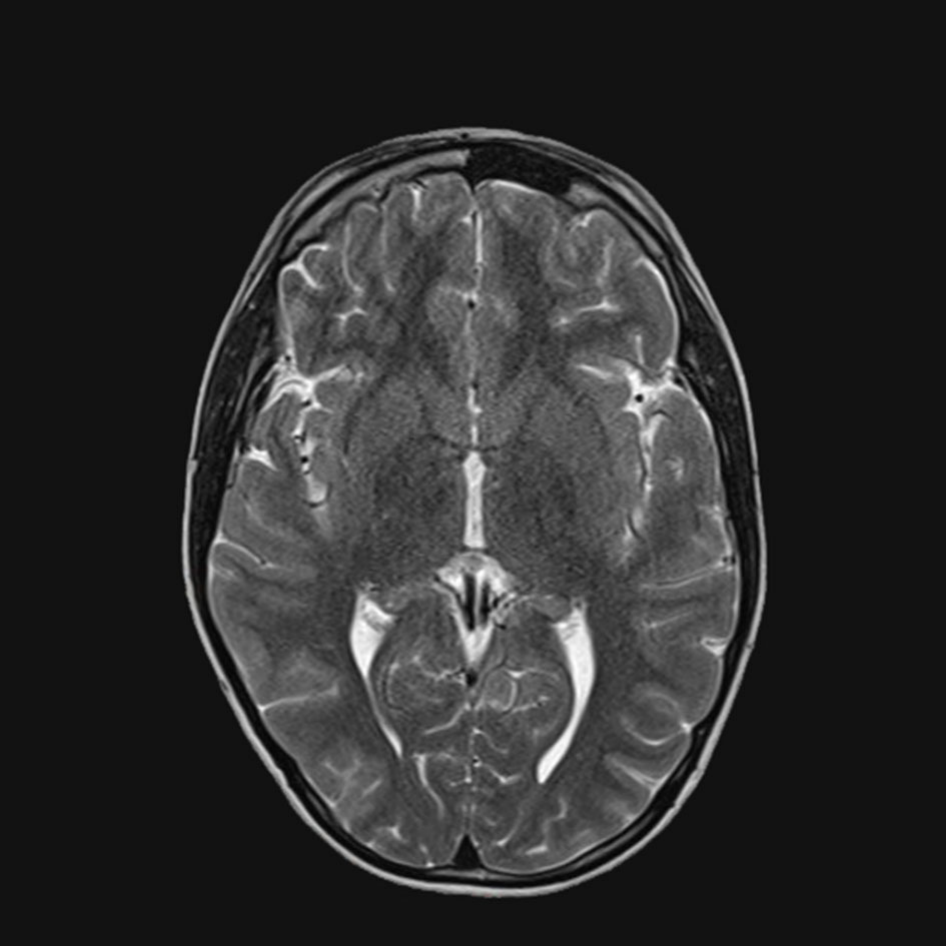
T2 MRI, brain axial image without alterations.

After the initial therapy with steroids and IVIG, no improvement was observed. Subsequently, plasma exchange therapy and rituximab infusion led to an important improvement that was documented in the brain and spinal cord MRI without contrast, performed 3 months later. At the spinal cord level, a reduction in alteration was observed (Figure 4), and no alterations were observed in the brain (Figures 5, 6). Clinically, the patient reported urinary complications of the neurogenic bladder. Unfortunately, paraplegia persisted, and the patient continued motor physiotherapy.

## Discussion

From the beginning of the COVID-19 pandemic, it was clear that even if the majority of symptoms involved the airway and pulmonary system, the disease would affect individuals at a multi-systemic level. In the first period, the cases among the pediatric population were not significant and seemed to have a milder effect globally, with minor cases of sequelae. With time and the increase in the number of children and adolescents affected by SARS-CoV-2, an opposite trend was observed. In particular, neurological manifestations, during or after the infection, are frequently diagnosed across all ages. This was evident even with differences in type and prevalence with respect to age, sex, and preexisting condition (10).

Neurological complications related to SARS-CoV-2 infection can involve the central nervous system (CNS) or the peripheral nervous system (PNS). These can be divided into specific complications (encephalopathy, seizures, stroke, meningitis, ataxia, ADEM, and Guillain–Barré syndrome) or non-specific complications (headache, myalgia, fatigue, and dizziness). From the time of infection onset to the beginning of neurological symptoms, we can differentiate between neurological involvement during COVID-19, after recovery from COVID-19, and during MIS-C (11). The different time with which complications can appear is probably linked to different pathogenetic mechanisms. The possibility of direct damage by the virus to nerve cells, probably through the invasion of the olfactory nerve and passage through the CNS, would explain the complications during the acute phase of COVID-19 infection. Damage to the vascular endothelia, and the interference with the ACE 2 enzyme, would seem responsible for a pro-inflammatory and pro-coagulant state that can cause complications even after acute COVID-19 infection. Furthermore, there are additional mechanisms such as an inflammatory and autoimmune injury that are responsible for the manifestations that appear days or weeks after the infection during convalescence (post-infectious inflammation) (1, 12, 13).

Risk factors and neurological complications in children and adolescents hospitalized with COVID-19 or MIS-C in the United States are described by La Rovere et al. Among them, 22% had neurological involvement. Although most of the cases displayed transient neurological signs, 12% of the patients developed a range of life-threatening conditions (severe encephalopathy, stroke, ADEM, acute fulminant cerebral edema, and GBS). Another 66% of these patients had unfavorable outcomes (death or persistent disability). The related risk factors were severe COVID-19 symptoms, underlying medical conditions (neurological, metabolic, congenital or genetic defects, malignancy, and premature transplantation), and MIS-C (4, 14). Sa et al. (15) reported that 12% of patients diagnosed with MIS-C presented neurological manifestations as altered consciousness, behavioral changes, focal neurological signs, persistent headache, hallucinations, and new onset of seizures, with the severity of symptoms, and their persistence in time associated with high levels of inflammatory blood index.

ADEM, also known as post-infectious encephalomyelitis and immune-mediated encephalomyelitis, is a multifocal and monophasic inflammatory demyelinating disease of the central nervous system (CNS) that involves multiple areas of the white matter of the brain and spinal cord and rarely the gray matter (6). It is mainly a pediatric disease and affects children, more often male children under 10 years, with a history of viral infection or vaccinations within 2–40 days or earlier (16). It is characterized by the acute onset of different neurological signs and symptoms, a prodromal phase (malaise, headache, nausea, vomiting), shortly followed by encephalopathy and meningeal signs, and rapidly progressive neurological deficits. There is a wide spectrum of severity from non-specific irritability, headache, and somnolence to rapid progression of symptoms and signs to coma, decerebrate rigidity, respiratory failure secondary to brainstem involvement, or severely impaired consciousness, which occurs in 11%−16% of cases (17).

In our patient, the ADEM diagnosis was formulated according to the consensus criteria of the International Pediatric Multiple Sclerosis Study Group in 2013: a polyfocal, clinical central nervous system (CNS) event with a presumed inflammatory demyelinating cause, an encephalopathy that cannot be explained by fever, no new clinical and MRI findings emerging 3 months or more after the onset, and abnormal brain MRI during the acute phase (18).

With regard to the fact that symptoms of neurological involvement appeared 12 days after SARS-CoV-2 infection, it is possible to presume that infection is the triggering cause of the immune-mediated response against CNS (12). The immune hyperactivity after COVID-19, as seen also during MIS-C, with high levels of inflammatory markers, imbalance in types, and the number of lymphocytes pro-inflammatory cytokines can disrupt the blood–brain barrier and activate glial cells, leading to neuronal functional disturbance, encephalopathy, loss of synapses, and even neuronal death (12), which can explain the severe course and the negative outcome of our patient.

Is important to note that anti-MOG antibodies can be present in 40%−68% of children with ADEM diagnosis (19). Myelin oligodendrocyte glycoprotein (MOG)-associated disorder (MOGAD) is a nervous system demyelination disease with various phenotypes including ADEM presentation (20, 21) with the presence of anti-MOG antibodies. The detection of MOG-IgG has been greatly improved by the cell-based assay test method (CBA) (20, 22). One limit of our case is that anti-MOG antibodies were measured by enzyme-linked immunosorbent assay, not considered the gold standard; it means that there is the possibility that also in our patients, anti-MOG was positive.

In addition, SARS-CoV-2 infection can be a possible trigger of MOGAD. The most common clinical presentations of MOGAD SARS-CoV-2-seropositive patients include optic neuritis and myelitis (23–25).

## Conclusion

As observed, the development of neurological symptoms during or after SARS-CoV-2 infection is common and may display a large spectrum of severity according to different risk factors. An interesting facet of the current study was that the patient acquired the COVID-19 infection without any symptoms. He did not have any underlying medical condition. However, his neurological signs and symptoms were dramatically severe and persistent that continued to persist. After almost months of the infection, the patient continued to have severe impairments.

This case invites us not to consider COVID-19 as a self-limiting mild infection. It strongly encourages us to be aware of the severe consequences that can occur during and after COVID-19 infection.

## Patient perspective

The patient was not aware of the gravity of the illness. He describes it as exhausting; the long hospitalization and the different treatments. Illness has changed his life with severe disability.

## Data availability statement

The original contributions presented in the study are included in the article/supplementary material, further inquiries can be directed to the corresponding author.

## Ethics statement

Written informed consent was obtained from the minor(s)' legal guardian/next of kin for the publication of any potentially identifiable images or data included in this article.

## Author contributions

FC and MF conceptualized and designed the case report, drafted the initial manuscript, and reviewed and revised the manuscript. LP and GM supervised and coordinated the manuscript and reviewed and revised the manuscript. AS, FM, and MV contributed to the conception and design of the manuscript, coordinated and supervised data collection, and critically reviewed the manuscript for important intellectual content. All authors have contributed to the manuscript and approved the submitted version.

## References

[1] Stafstrom CE. Neurological effects of COVID-19 in infants and children. Dev Med Child Neurol. (2022) 64:818–29. 10.1111/dmcn.15185 35243616 PMC9111795

[2] UNICEF DATA ON LINE. Available online at: https://data.unicef.org/resources/covid-19-confirmed-cases-and-deaths-dashboard/ (accessed October 7, 2022).

[3] Dong Y Mo X Hu Y Qi X Jiang F Jiang Z. Epidemiology of COVID-19 among children in China. Pediatrics. (2020) 145:e20200702. 10.1542/peds.2020-0702 32179660

[4] LaRovere KL Riggs BJ Poussaint TY Young CC Newhams MM Maamari M. Neurologic involvement in children and adolescents hospitalized in the United States for COVID-19 or multisystem inflammatory syndrome. JAMA Neurol. (2021) 78:536–47. 10.1001/jamaneurol.2021.0504 33666649 PMC7936352

[5] Ray STJ Abdel-Mannan O Sa M Fuller C Wood GK Pysden K. Neurological manifestations of SARS-CoV-2 infection in hospitalised children and adolescents in the UK: a prospective national cohort study. Lancet Child Adolesc Health. (2021) 5:631–41. 10.1016/S2352-4642(21)00193-0 34273304 PMC8279959

[6] Esposito S Di Pietro GM Madini B Mastrolia MV Rigante D. A spectrum of inflammation and demyelination in acute disseminated encephalomyelitis (ADEM) of children. Autoimmun Rev. (2015) 14:923–9. 10.1016/j.autrev.2015.06.002 26079482 PMC7105213

[7] Kania K Ambrosius W Tokarz Kupczyk E Kozubski W. Acute disseminated encephalomyelitis in a patient vaccinated against SARS-CoV-2. Ann Clin Transl Neurol. (2021) 8:2000–3. 10.1002/acn3.51447 34480527 PMC8528462

[8] Kacmaz E Bozan G Carman KB Kilic O Arslanoglu MO Toprak U. Rituximab treatment in acute disseminated encephalomyelitis associated with *Salmonella* infection. Case Rep Pediatr. (2021) 2021:5570566. 10.1155/2021/5570566 33954003 PMC8068525

[9] Valderas C Méndez G Echeverría A Suarez N Julio K Sandoval F. COVID-19 and neurologic manifestations: a synthesis from the child neurologist's corner. World J Pediatr. (2022) 18:373–82. 10.1007/s12519-022-00550-4 35476245 PMC9044375

[10] Dewanjee S Vallamkondu J Kalra RS Puvvada N Kandimalla R Reddy PH. Emerging COVID-19 neurological manifestations: present outlook and potential neurological challenges in COVID-19 pandemic. Mol Neurobiol. (2021) 58:4694–715. 10.1007/s12035-021-02450-6 34169443 PMC8224263

[11] Siracusa L Cascio A Giordano S Medaglia AA Restivo GA Pirrone I. Neurological complications in pediatric patients with SARS-CoV-2 infection: a systematic review of the literature. Ital J Pediatr. (2021) 47:123. 10.1186/s13052-021-01066-9 34078441 PMC8170632

[12] Lin JE Asfour A Sewell TB Hooe B Pryce P Earley C. Neurological issues in children with COVID-19. Neurosci Lett. (2021) 743:135567. 10.1016/j.neulet.2020.135567 33352286 PMC7831718

[13] Jha NK Ojha S Jha SK Dureja H Singh SK Shukla SD. Evidence of coronavirus (CoV) pathogenesis and emerging pathogen SARS-CoV-2 in the nervous system: a review on neurological impairments and manifestations. J Mol Neurosci. (2021) 71:2192–209. 10.1007/s12031-020-01767-6 33464535 PMC7814864

[14] Fink EL Robertson CL Wainwright MS Roa JD Lovett ME Stulce C. Prevalence and risk factors of neurologic manifestations in hospitalized children diagnosed with acute SARS-CoV-2 or MIS-C. Pediatr Neurol. (2022) 128:33–44. 10.1016/j.pediatrneurol.2021.12.010 35066369 PMC8713420

[15] Sa M Mirza L Carter M Carlton Jones L Gowda V et. Systemic inflammation is associated with neurologic involvement in pediatric inflammatory multisystem syndrome associated with SARS-CoV-2. Neurol Neuroimmunol Neuroinflamm. (2021) 8:e999 10.1212/NXI.0000000000000999 33850037 PMC8054962

[16] Tenembaum S Chitnis T Ness J Hahn JS International Pediatric MS Study Group. Acute disseminated encephalomyelitis. Neurology. (2007) 68(16 Suppl 2):S23–36. 10.1212/01.wnl.0000259404.51352.7f 17438235

[17] Wingerchuk DM. Postinfectious encephalomyelitis. Curr Neurol Neurosci Rep. (2003) 3:256–64. 10.1007/s11910-003-0086-x 12691631

[18] Krupp LB Tardieu M Amato MP Banwell B Chitnis T Dale RC. International Pediatric Multiple Sclerosis Study Group criteria for pediatric multiple sclerosis and immune-mediated central nervous system demyelinating disorders: revisions to the 2007 definitions. Mult Scler. (2013) 19:1261–7. 10.1177/1352458513484547 23572237

[19] Ambrosius W Michalak S Kozubski W Kalinowska A. Myelin oligodendrocyte glycoprotein antibody-associated disease: current insights into the disease pathophysiology, diagnosis and management. Int J Mol Sci. (2020) 22:100. 10.3390/ijms22010100 33374173 PMC7795410

[20] Li Y Liu X Wang J Pan C Tang Z. Clinical features and imaging findings of myelin oligodendrocyte glycoprotein-IgG-associated disorder (MOGAD). Front Aging Neurosci. (2022) 14:850743. 10.3389/fnagi.2022.850743 35370624 PMC8965323

[21] Shahriari M Sotirchos ES Newsome SD Yousem DM. MOGAD: how it differs from and resembles other neuroinflammatory disorders. AJR Am J Roentgenol. (2021) 216:1031–9. 10.2214/AJR.20.24061 32755221

[22] Reindl M Schanda K Woodhall M Tea F Ramanathan S Sagen J. International multicenter examination of MOG antibody assays. Neurol Neuroimmunol Neuroinflamm. (2020) 7:e674. 10.1212/NXI.0000000000000674 32024795 PMC7051197

[23] Mariotto S Carta S Dinoto A Lippi G Salvagno GL Masin L. Is there a correlation between MOG-associated disorder and SARS-CoV-2 infection? Eur J Neurol. (2022) 29:1855–8. 10.1111/ene.15304 35224824 PMC9111815

[24] Lambe J McGinley MP Moss BP Mao-Draayer Y Kassa P Ciotti JR et al. Myelin oligodendrocyte glycoprotein-IgG associated disorders (MOGAD) following SARS-CoV-2 infection: a case series. J Neuroimmunol. (2022) 370:577933. 10.1016/j.jneuroim.2022.577933 35878436 PMC9279254

[25] Johnsson M Asztely F Hejnebo S Axelsson M Malmeström C Olausson T. SARS-CoV-2 a trigger of myelin oligodendrocyte glycoprotein-associated disorder. Ann Clin Transl Neurol. (2022) 9:1296–301. 10.1002/acn3.51609 35713508 PMC9349599

